# Modalities and Mechanisms of Treatment for Coronavirus Disease 2019

**DOI:** 10.3389/fphar.2020.583914

**Published:** 2021-02-08

**Authors:** Zhihong Zuo, Ting Wu, Liangyu Pan, Chenzhe Zuo, Yingchuo Hu, Xuan Luo, Liping Jiang, Zanxian Xia, Xiaojuan Xiao, Jing Liu, Mao Ye, Meichun Deng

**Affiliations:** ^1^ Department of Biochemistry and Molecular Biology and Hunan Province Key Laboratory of Basic and Applied Hematology, School of Life Sciences, Central South University, Changsha, China; ^2^ Xiangya School of Medicine, Central South University, Changsha, China; ^3^ Department of Cardiovascular Medicine, The Third Xiangya Hospital, Central South University, Changsha, China; ^4^ Hunan Yuanpin Cell Biotechnology Co., Ltd., Changsha, China; ^5^ Department of Cell Biology, School of Life Sciences, Central South University, Changsha, China; ^6^ Hunan Key Laboratory of Animal Models for Human Diseases, Hunan Key Laboratory of Medical Genetics and Center for Medical Genetics, School of Life Sciences, Central South University, Changsha, China; ^7^ Molecular Science and Biomedicine Laboratory, State Key Laboratory for Chemo/Biosensing and Chemometrics, College of Biology, College of Chemistry and Chemical Engineering, Collaborative Innovation Center for Molecular Engineering for Theranostics, Hunan University, Changsha, China

**Keywords:** SARS-CoV-2, COVID-19, mechanisms, treatment, therapeutic agents

## Abstract

Coronavirus disease 2019 (COVID-19), which is caused by severe acute respiratory syndrome coronavirus 2 (SARS-CoV-2), is spreading rapidly throughout the world. Although COVID-19 has a relatively low case severity rate compared to SARS and Middle East Respiratory syndrome it is a major public concern because of its rapid spread and devastating impact on the global economy. Scientists and clinicians are urgently trying to identify drugs to combat the virus with hundreds of clinical trials underway. Current treatments could be divided into two major part: anti-viral agents and host system modulatory agents. On one hand, anti-viral agents focus on virus infection process. Umifenovir blocks virus recognizing host and entry. Remdesivir inhibits virus replication. Chloroquine and hydroxychloroquine involve preventing the whole infection process, including virus transcription and release. On the other hand, host system modulatory agents are associated with regulating the imbalanced inflammatory reaction and biased immune system. Corticosteroid is believed to be commonly used for repressing hyper-inflammation, which is one of the major pathologic mechanisms of COVID-19. Convalescent plasma and neutralizing antibodies provide essential elements for host immune system and create passive immunization. Thrombotic events are at high incidence in COVID-19 patients, thus anti-platelet and anti-coagulation are crucial, as well. Here, we summarized these current or reproposed agents to better understand the mechanisms of agents and give an update of present research situation.

## Introduction

The coronavirus disease 2019 (COVID-19) pandemic, which is caused by the severe acute respiratory syndrome coronavirus 2 (SARS-CoV-2), has now affected 210 countries and territories, with more than five million confirmed cases. COVID-19 is rapidly spreading around the world, leading to widespread public concern and a global response. SARS-CoV-2, along with severe acute respiratory syndrome coronavirus (SARS-CoV) and Middle East respiratory syndrome coronavirus (MERS-CoV), belong to the beta-coronavirus family. The full-length genome sequence of SARS-CoV-2 is 79.5% similar to MERS-CoV and 50% similar to SARS-CoV (Zhou P et al., 2020). Although we have learned much about the etiology and molecular characteristics of SARS-CoV-2, the origin of this novel virus remains unclear. Many studies support the hypothesis that bats are the most likely original host, with other animals, such as snakes or minks, acting as intermediate hosts (Li et al., 2005; Guo et al., 2020; Ji et al., 2020). SARS-CoV-2 targets the respiratory tract, but the lack of specific early symptoms makes it difficult to distinguish COVID-19 from other respiratory infections. Fever, cough, fatigue, and dyspnea are the most common early symptoms of COVID-19 (Booth et al., 2003; Yang J et al., 2020). In European patients, olfactory and gustatory dysfunction may precede the onset of respiratory symptoms and can be significant (Lechien et al., 2020). Patients with severe COVID-19 are vulnerable to complications and multi-organ damage (Huang C et al., 2020; Wu T et al., 2020). Compared with SARS and MERS, COVID-19 has a lower case-fatality, but the virus has a higher basic reproduction number and higher transmissibility (Chan et al., 2003; Zumla et al., 2015). SARS-CoV-2 can be spread within communities, households, and hospitals by confirmed COVID-19 patients or by asymptomatic individuals (Zhang X et al., 2020). The predominant transmission routes are droplet transmission and close contact, although other transmission routes such as erosol and fecal-oral transmission are possible, but not confirmed or refuted. It has been suggested that each patient with COVID-19 infects approximately 2.2 close contacts (Li Q et al., 2020), which partially accounts for the global COVID-19 pandemic.

Although some potential strategies for preventing the infection are proposed (Kang et al., 2020), however, in the absence of an effective vaccine, identification of effective drugs is crucial to treatment of this novel coronavirus. Both clinical experience and exploratory studies with other coronaviruses suggest more than 20 agents that may be potentially used to treat COVID-19. Some of these drugs such as corticosteroids, Chloroquine and Hydroxychloroquine (CQ/HCQ), as well as Lopinavir and Ritonavir (LPV/r) have been widely used in clinical practice, whereas others, such as Janus Kinase (JAK) inhibitors, have been introduced only recently. In this review, we have compiled all available evidence with which to establish a framework for COVID-19 treatment as well as therapeutic optimization.

## Mechanisms of Virus Infection

Recognition: SARS-CoV-2 is a positive-stranded ribonucleic acid (+RNA) virus, whose genes encode 16 nonstructural proteins (nsp1 to nsp16) and four structural proteins, including Membrane (M), Spike(S), Envelope (E) and Nucleocapsid (N). Among them, S protein makes contribution to homo-trimeric spikes which are responsible for the virus entry via recognizing with the host receptor angiotensin converting enzyme II (ACE2) (Chen Y et al., 2020). S proteins can be cleaved into by an appropriate protease into two functional domains (S1 and S2) (Hoffmann et al., 2020). The receptor-binding domain (RBD) within S1 subunit is a key functional component for binding with ACE2 (Lan et al., 2020). In addition, S1 can be further divided into a C-terminal domain (CTD) and N-terminal domain (NTD). In contrast with MERS-CoV and SARS-CoV, SARS-CoV-2 applies the S1 CTD to interact with ACE2 (Wang Q et al., 2020). It is reported that the combination of spikes and ACE2 promotes the dissociation of the S1 with ACE2, which results in the transition of S2 to mediate fusion with cell membrane (Gui et al., 2017). The role of ACE2 in mediating entry of the virus also is highlighted (Hoffmann et al., 2020; Zhou P et al., 2020). Binding of virus to ACE2 is an important initiation of viral infections, thus any drugs prevent the process can be identified as a treatment option for COVID-19. Convalescent plasma (CP) and immunoglobins (IG) collected from recovery COVID-19 patients contain neutralizing antibodies, which could bind to S1-RBD, inhibiting the binding of virus with receptor, thus limiting viral entry (Rojas et al., 2020). Umifenovir fights against SARS-CoV-2 effectively by blocking or hindering trimerization of S protein (Vankadari, 2020). CQ/HCQ might inhibit entry process by interfering with the glycosylation of ACE2 and CQ also possesses the ability to inhibit sialic acid, which significantly affects activity of ACE2 (Kwiek et al., 2004; Hashem et al., 2020).

Entry: Coronavirus enter the host cells through two pathways: the endocytosis or membrane fusion. During the endocytosis, the viruses engulfed into a double-membrane structure firstly enter the early endosomes, and then they are mainly delivered to the late endosome, followed by fusing with lysosome. Within lysosome, the S protein undergoes a series of modifications and enzymatic cleavages, and then viral RNA is released into cytoplasm (Yang and Shen, 2020). Notably, the process is highly pH-dependent and acidic environment is required. However, CQ/HCQ might neutralize their pH by accumulating in endosomes and lysosomes (Hashem et al., 2020; Wang M et al., 2020). Umifenovir is involved in the inhibition of membrane fusion of the viral envelope and host cell membrane (Kadam and Wilson, 2017).

RNA Replication: Coronavirus replicate the virus genomes by making use of the materials of host cells. After releasing of virus RNA into cytoplasm, the ribosomes of host cells are used to produce polyproteins, which are subsequently cleaved into smaller molecules applying for replicating new viruses by enzymes, including 3-Chymotrypsin like protease (3CLpro) and the papain-like protease (PLpro). In addition, an RNA-dependent RNA polymerase (RdRp) is expressed to generate the complementary RNA strand using the virus RNA as a template (Mullard, 2018; Huang J et al., 2020; Wrapp et al., 2020). RdRp is an essential enzyme for coronavirus replication, providing a new insight for the antiviral agents for COVID-19 treatment. Remdesivir can bind with RdRp, thus RdRp is unable to incorporate RNA subunits, resulting in prevention of virus genome replication (Tchesnokov et al., 2019; Elfiky, 2020a). In addition, Ribavirin, Favipiravir and HCQ are thought to have the ability to interact with RdRp active site (Elfiky, 2020b). Zinc salts inhibits RdRp and has been shown to against coronavirus (te Velthuis et al., 2010). LPV/r have been found to tightly bind to the active sites of SARS-CoV-2 3CLpro, inhibiting the replication of new viruses (Nutho et al., 2020).

Transcription and release: A series of sub-genomic mRNAs are produced by discontinuous transcription and then are translated into related viral proteins. The envelope glycoproteins are newly formed and inserted into the membrane of the endoplasmic reticulum (ER) or Golgi, and the nucleocapsid consists of genomic RNA and nucleocapsid protein. Then, viral particles containing viral proteins and genome RNA can be budded into the ER-Golgi intermediate compartment. Finally, they are transported through vesicles and released out of the cell by exocytosis (Li X et al., 2020). CQ/HCQ can suppress the post-translational modification of viral proteins, which occur within the ER or *trans*-Golgi network (Savarino et al., 2004). The process of assembly and budding can be interfered by CQ/HCQ with accumulation of viral vesicles in *trans*-Golgi network (Harley et al., 2001).

Cytokine storm: Like other viral infection, cytokines play an essential role in the progression of COVID-19. Higher levels of cytokines, including granulocyte-macrophage colony stimulating factor, monocyte-chemokine protein 1, interferon-inducible protein-10 and tumor necrosis factor-α (TNF-α), were more commonly seen in patients with severe COVID-19 than in those with non-severe COVID-19, suggesting cytokine profiles are closely associated with COVID-19 severity (Huang C et al., 2020). The level of interleukin (IL)-2R, IL-1 and IL-6 in serum can be significantly predictors of the severity of patients with COVID-19 (Chen L et al., 2020). In addition, pathological examination of biopsy samples demonstrate that inflammatory cellular infiltration is common in multiple organs, including the lung, heart, kidney, and liver (Tian S et al., 2020; Xu Z et al., 2020). This suggests that viruses aggravate the indirect injury through proinflammatory function or cytokine storms. Therefore, monoclonal antibodies or agents targeting different cytokine also represent attractive therapeutic options for COVID-19. It is well-known that corticosteroid, CP and IG are supposed to inhibit cytokine storms and modulate dysfunctional immune system (Amoss and Chesney, 1917; McGuire and Redden, 1918; Winkler and Koepsell, 2015). By preventing or attenuating the cytokine storm by secreting powerful anti-inflammatory factors, mesenchymal stem cells (MSCs) could also, theoretically, suppress overreaction of the immune system (Alhazzani et al., 2020). Various pro-inflammatory cytokines, including IL-1, IL-6 and TNF-α can be reduced by the CQ/HCQ (Schrezenmeier and Dörner, 2020). Common anti-TNF agents, such as infliximab, adalimumab, thalidomid and golimumab, are believed to combat cytokine release syndrome, since TNF is a vital intermediated factor in the cytokine storm (Mitoma et al., 2018). Anakinra, an IL-1 receptor antagonist that blocks cytokine release, is used to treat inflammation-related diseases and can be beneficial for treating severe COVID-19 patients (Pasi et al., 2015). Tocilizumab and eculizumab (Davies and Choy, 2014) are monoclonal antibodies against IL-6 and the complement protein C5 reverse the cytokine storm respectively and improve the condition of severely COVID-19 patients.

## Anti-viral Agents

### Hydroxychloroquine and Chloroquine

**FIGURE 1:**
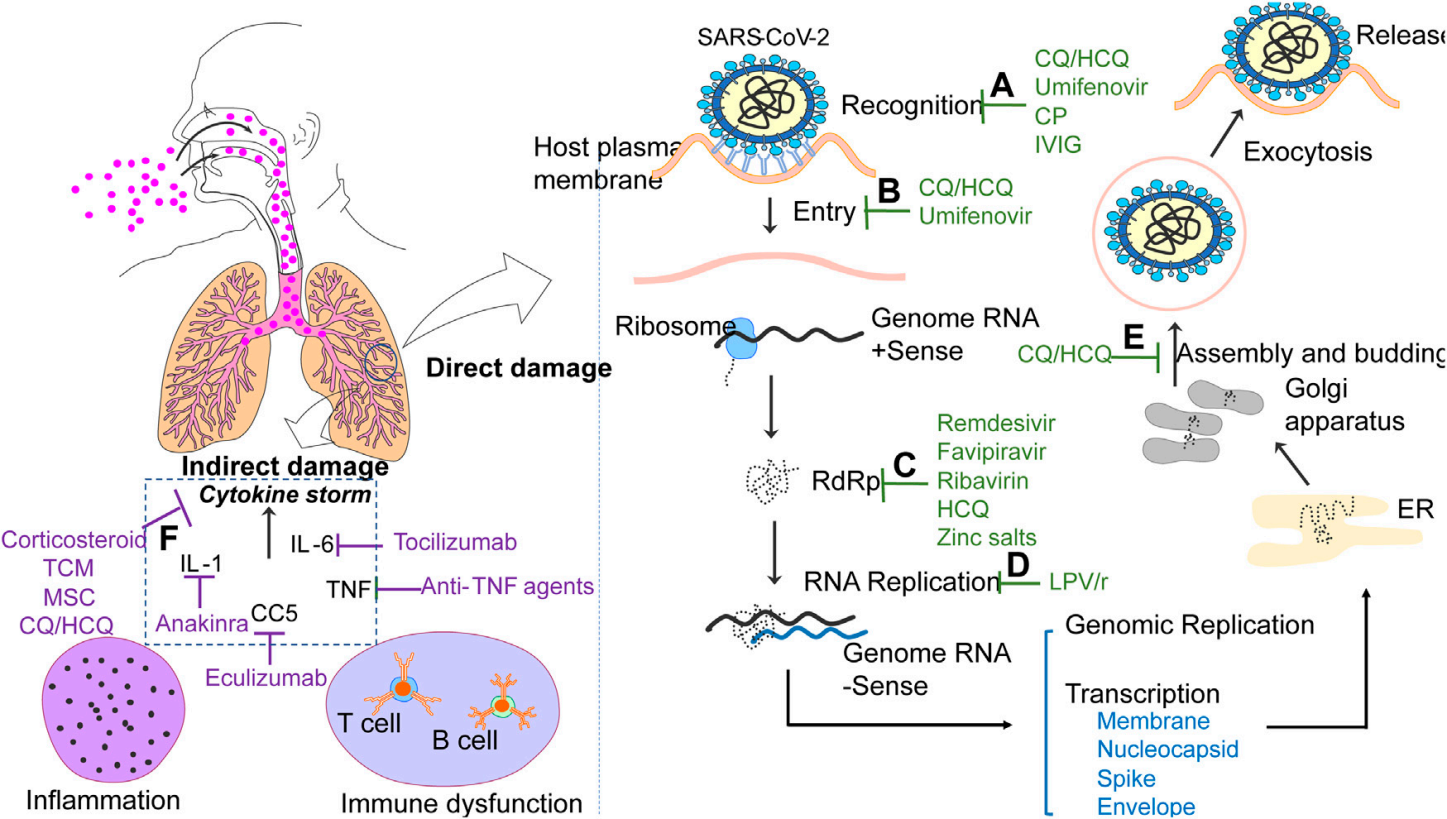
Cellular and molecular possible sites of action of agents for COVID-19 treatment. 1. CP and IVIG inhibit the binding of virus with receptor by interacting with S1-RBD. Umifenovir interfere the recognition by blocking or hindering trimerization of S protein, while CQ/HCQ interferes with the activity of ACE2. 2. CQ/HCQ inhibit virus pH-dependent endocytosis through increasing pH. Umifenovir is also involved in the inhibition of membrane fusion. 3. Remdesivir, Ribavirin, Favipiravir, HCQ and Zinc salts can bind with RdRp, resulting in prevention of virus genome replication. 4. LPV/r inhibit the replication of new viruses by tightly binding to the active sites of virus3CLpro 5. CQ/HCQ suppress the assembly and budding of virus. 6. Treatment strategies reduce tissues damage by targeting various cytokine storms.

CQ/HCQ exerted inhibitory effects from recognition process to cytokine storm production (Figures 1A–F). There are many clinical trials around the world including in China (Cortegiani et al., 2020). A RCT showed that high CQ dosage should not be recommended for critically ill patients with COVID-19 because of its potential safety hazards (Borba et al., 2020). Low dose of HCQ (200 mg twice a day for 7–10 days) reduces fatality of critically ill patients with COVID-19. In France, a clinical trial showed that HCQ significantly reduced viral load in patients infected with SARS-CoV-2, especially when co-administered with azithromycin (Gautret et al., 2020a), which was supported by the conclusion of another study (Gautret et al., 2020b). Furthermore, a clinical trial showed that CQ may have a slight advantage over LPV/r in combating SARS-CoV-2 (Huang M et al., 2020). However, the results of recent studies against these promising conclusions. Though, HCQ was reported to promote viral load reduction/disappearance in COVID-19 patients and the effect was reinforced by azithromycin, it is limited by the sample size (Gautret, et al., 2020a). A study showed no significantly reduced requirement for mechanical ventilation or decreased overall mortality in patients treated with HCQ (Magagnoli et al., 2020), another research didn’t support its use in patients admitted to hospital with COVID-19 who require oxygen, either (Vinetz, 2020). Treatment with HCQ, azithromycin, or both, compared with neither treatment, was not significantly associated with differences in in-hospital mortality among patients with COVID-19 (Rosenberg et al., 2020). No evidence supported the beneficial effects of application of HCQ for COVID-19 patients who require oxygen (Mahévas et al., 2020). In terms of viral RNA clearance, administration of HCQ did not result in a significantly higher probability of negative conversion than standard of care alone in patients with mainly persistent mild to moderate COVID-19 (Tang W et al., 2020). Neither a multi-center RCT nor a retrospective study demonstrated HCQ shorten viral shedding in non-severe COVID-19 patients (Chen C P et al., 2020). Even in mild, early stage outpatients, HCQ did not substantially reduce symptom severity (Skipper et al., 2020). It was not associated with either a greatly lowered or an increased risk of the composite end point of intubation or death (Geleris et al., 2020). Notably, clinicians should pay more attention to the adverse effects caused by CQ/HCQ. CQ/HCQ showed retinal toxicity after long-term use for systemic lupus erythematosus and other rheumatoid diseases but some researchers believe the likelihood of retinal damage in COVID-19 patients seems to be extremely low because the dose is 3–4-fold lower than the normal dose and the duration of treatment is much shorter (Marmor, 2020). CQ/HCQ have also been associated with QT interval prolongation (Mercuro et al., 2020) and may thus lead to cardiac arrests (Lecuit, 2020), so QT interval should be followed repeatedly in patients with COVID-19 who are treated with HCQ/AZ (Chorin et al., 2020). A study of case series revealed key limitations, which include a potential lack of generalizability beyond the ICU, because of cardiac complication (Bessière et al., 2020). The cardiac safety profile may, however, be ameliorated by using single enantiomers of CQ/HCQ (Lentini et al., 2020). Serious cutaneous adverse reactions, fulminant hepatic failure and other side effects have also been reported (Ferner and Aronson, 2020). Because CQ has also been shown to reduce glucose-6-phosphate dehydrogenase (G6PD) activity, care should be taken when administering HCQ and CQ to G6PD-deficient patients, who may be more susceptible to SARS-CoV-2 (Kassi et al., 2020). The known and potential benefits of chloroquine and hydroxychloroquine no longer outweigh the known and potential risks, so that FDA revoked the emergency use. Recently, HCQ is further proposed as postexposure prophylaxis, unfortunately, two studies showed that it could not prevent SARS-CoV-2 infection (Boulware et al., 2020; Mitja et al., 2020). Basically, conflicting conclusion from current research, high possibility of adverse effects and lack of clinical trials in large population restrict CQ/HCQ use.

### Remdesivir

Remdesivir fights against virus by interacting with RdRp active site to inhibit virus replication (Figure 1C). In view of it antiviral capacity, researches show significant *in vitro* activity of remdesivir against different viruses, including Ebola virus, Paramyxoviridae, Pneumoviridae (Lo et al., 2017) and many coronaviruses (Sheahan et al., 2017). Administration of remdesivir in both animal model and patients with Ebola showed amelioration of symptoms (Jacobs et al., 2016; Warren et al., 2016). Hence, the clinical potential of remdesivir is now being re-examined by clinicians as a result of the current SARS-CoV-2 pandemic (Ko et al., 2020). In the first confirmed case of SARS-Cov-2 in the United States, the patient’s oropharyngeal swab tested turned negative after administration of remdesivir for 6 days (Holshue et al., 2020). Remdesivir can also benefit patients with SARS-CoV-2 pneumonia hospitalized outside ICU where clinical outcome was better and adverse events are less frequently observed (Antinori et al., 2020). Additionally, a simulated two-arm controlled study corroborated the efficacy of remdesivir, including reducing death, increasing rate of discharge (Hsu et al., 2020). Compared to placebo group, remdesivir accelerates recovery in adult patients and decreased respiratory tract infection rate (Beigel et al., 2020). Remdesivir was related to significantly greater recovery and 62% reduced odds of death vs. standard-of-care treatment in severe patients (Olender et al., 2020). A special population, pregnant and postpartum women, with severe COVID-19 receiving compassionate use remdesivir, got high recovery rates and were at a low-risk suffering from serious adverse events (Burwick et al., 2020). However, a 5-days course and a 10-days course of remdesivir did not make any difference in patients with severe Covid-19 not requiring mechanical ventilation (Goldman et al., 2020). A RCT indicated difference of clinical status between a 5-days course of remdesivir and standard care was of uncertain clinical importance (Spinner et al., 2020). More disappointingly, accordingly to a clinical trial, compared with placebo group, remdesivir neither speeded up recovery nor reduced death in COVID-19 patients, but the true effectiveness was uncovered by lack of new enrolled patients in Wuhan (Grein et al., 2020). A randomized, double-blind, placebo-controlled, multicenter trial showed remdesivir was not associated with statistically significant clinical benefits among adult patients admitted to hospital for severe COVID-19. However, the numerical reduction in time to clinical improvement in those treated earlier requires confirmation in larger studies (Wang Y et al., 2020). By the way, one note of caution is that high doses of remdesivir may induce testicular toxicity and result in deterioration of sperm parameters in mice (Fan et al., 2020). Given the low certainty evidence for critical outcomes and promising faster clinical improvement for the treatment of SARS-CoV-2, use of remdesivir is weakly recommended. Ongoing trials, together with further randomized controlled trials following ethical approval, are needed to fully evaluate the efficacy and safety of remdesivir.

### Lopinavir and Ritonavir

LPV/r inhibits viral replication by binding to the active sites of SARS-CoV-2 3CLpro (Figure 1D). For SARS-CoV-2, LPV/r shows promising prospect based on clinical investigations. A report of case series showed significant signs of improvement in pneumonia-associated symptoms after antiviral treatment including LPV/r (Wang Z et al., 2020). Other reported cases described decreased viral load and clinical improvement after LPV/r administration (Han et al., 2020; Li Y et al., 2020a; Lim et al., 2020; Wang Z et al., 2020). Earlier administration of LPV/r treatment could shorten viral shedding (Yan et al., 2020). Compared with adjuvant drugs alone, the combination of adjuvant drugs and LPV/r could lower the body temperature and restore normal physiological mechanisms with no evident toxic and side effects (Ye X T et al., 2020). Triple combination of interferon beta-1b, LPV/r and ribavirin were safe and superior to LPV/r alone in alleviating symptoms and shortening the duration of viral shedding and hospital stay in patients with mild to moderate COVID-19 (Hung et al., 2020). However, the 28-days mortality of severe COVID-19 patients treated with LPV/r was similar with those of patients in the standard care group (Cao B et al., 2020; Stower, 2020). A randomized trial concluded that LPV/r was not associated with hospital stay, or risk of progressing to invasive mechanical ventilation or death, which do not support use for treatment of patients admitted to hospital with COVID-19 (RECOVERY Collaborative Group, 2020). In addition, the use of LPV/r was even associated with delayed clearance of viral RNA (Chen X et al., 2020). In a study carried out in Singapore, however, four out of five patient (80%) developed nausea, vomiting, and/or diarrhea, which precluded completion of the planned 14-days treatment course (Young et al., 2020). Severe jaundice was more frequently observed in patients treated with LPV/r (Levy et al., 2020). Based on pharmacokinetics, it is difficult to recommend oral LPV/r safe dose without compromising the benefit of the antiviral strategy (Lê et al., 2020). At present, the effectiveness and safety of LPV/r have not yet been confirmed due to controversial results, thus more clinical evidence is required for further evaluation of efficiency and safety.

### Favipiravir

Favipiravir, which could interfere with the action of RdRp (Figure 1D), was reported to be effective in reducing SARS-CoV-2 infection *in vitro* (Wang M et al., 2020). A clinical study showed that treatment with FPV was safe and had no severe adverse effects. It also improved chest CT scans and viral clearance in patients with COVID-19, with a 4-days viral clearance time for FPV vs. an 11-days time for the control group (Cai et al., 2020), although the 7-days clinical recovery rate remains controversial (Chen C et al., 2020). It was also found that FPV significantly improved treatment effects on COVID-19 in terms of disease progression and viral clearance (Cai et al., 2020). Yet, another prospective study suggested viral clearance measured by RT-PCR by day 6 was not significantly advanced but FPV reduced time to defervescence (Doi et al., 2020). Addition of FPV into existing standard treatment was not proved to be beneficial, either (Lou et al., 2020). Oral administration of FPV even delayed viral clearance according a case series (Fu et al., 2020) and the adverse effect, fever, was firstly reported in two cases (Takoi et al., 2020). Favipiravir has been approved by the National Medical Products Administration of China as the first anti-COVID-19 drug in China, as the clinical trial had demonstrated efficacy with minimal side effects.

### Umifenovir

Umifenovir was involved in the prevention of recognition and the inhibition of membrane fusion to fight against virus (Figure 1B). It is shown *in vitro* that umifenovir reduced replication of SARS-CoV-2 compared with the control group, and the inhibition occurred efficiently at both viral entry and post-entry stages (Wang X et al., 2020). However, little benefit of umifenovir monotherapy was presented for improving the clinical outcome of mild/moderate COVID-19 patients’ over supportive care. This clinical trial involving 86 patients with mild COVID-19 found that the average time for SARS-CoV-2 positive-to-negative conversion in the umifenovir group was similar to that in the control group (Li Y et al., 2020a; Li Y et al., 2020b). There were also no significant differences in symptoms, or chest CT scans between the umifenovir and favipiravir groups, suggesting that umifenovir is less suitable for first-line treatment (Chen C et al., 2020). Patients in the umifenovir group had a shorter duration of positive RNA test compared to those in the lopinavir/ritonavir group (Zhu et al., 2020). However, a recent retrospective study indicated umifenovir might not improve the prognosis or accelerate SARS-CoV-2 clearance in non-ICU patients (Lian et al., 2020). Debating of umifenovir treatment strategy needs more evidence from clinical trials.

### Ivermectin

Ivermectin is a specific inhibitor of importin-α/β-dependent nuclear transport and shows antiviral potential against several RNA viruses by blocking the nuclear localization of viral proteins (Lv et al., 2018). It exerts antivirus effects toward both HIV-1 and dengue virus (DENV) with respect to the HIV-1 integrase and non-structural protein 5 (NS5) polymerase proteins, respectively (Wagstaff et al., 2012). Ivermectin can also dissociate the preformed host importin (IMP) α/β1 heterodimer, as well as prevent its formation (Rogers T F et al., 2020). These inhibitory effects coincide with the onset of intracellular viral RNA synthesis, as expected for a molecule that specifically targets the viral helicase (Mastrangelo et al., 2012). Based on its inhibition of RNA virus, Leon et al. suggested that treatment with ivermectin reduced cell-associated SARS-CoV-2 viral RNA by 93% after 24 h and by 99.8% after 48 h, with a single treatment achieving ∼5000-fold reduction in viral load (Caly et al., 2020). In patients who required higher inspired oxygen or ventilatory support, ivermectin application during treatment was associated with lower mortality (Rajter et al., 2020). As an add-on therapy, ivermectin was helpful for better effectiveness, shorter hospital-stay and relatively safe (Gorial et al., 2020). However, the approved dose of ivermectin was found to exert impact *in vitro*, which was challenged by a new research. To achieve an efficient plasma concentration, ivermectin use would be over 10 times higher than the approved dose, possibly resulting in adverse events (Schmith et al., 2020). The optimal dose and combination strategy have not been decided so far and require more evidence from clinical studies.

### Galidesivir

Galidesivir, an adenosine analogue, shows broad-spectrum antiviral activity against a wide range of RNA viruses and is under clinical development for treatment of Ebola and yellow fever virus infections. It mainly inhibits viral RdRp function, acting as a non-obligate RNA chain terminator (Warren et al., 2014). Galidesivir has been shown to treat several RNA viruses, such as Zika virus, and Rift Valley Fever virus, both *in vitro* and in animal model (Julander et al., 2017; Westover et al., 2018). For SARS-CoV-2, both a molecular docking study and an in silico perspective demonstrated Galidesivir can tightly bind to the RdRp of the SARS-CoV-2 strain and thus may be applied to treat the disease (Elfiky, 2020a; Elfiky, 2020b). However, *ex vivo* or *in vivo* experiment on COVID-19 is still lacking.

### Nelfinavir

Nelfinavir, a well-known HIV-1 protease inhibitor, is widely prescribed as part of triple-drug combination therapy for the treatment of HIV infection. Recently, this agent also exerted inhibition on the cytopathic effect induced by SARS-CoV infection, and suppressed replication of the SARS-CoV at the post-entry step of infection (Yamamoto et al., 2004), but nelfinavir did not exhibit activity against MERS-CoV *in-vitro* (Chan et al., 2013). Several virtual screening and mocking study indicated that nelfinavir was supposed to be a potential inhibitor of COVID-19 main protease (Duan et al., 2020a; Mittal et al., 2020; Ohashi et al., 2020). In the SARS-CoV-2 research, nelfinavir mesylate might bind inside the S trimer structure, which is proximal to the S2 amino terminus, then directly inhibit Sn and So-mediated membrane fusion. This drastically inhibited S protein-mediated cell fusion with complete inhibition (Musarrat et al., 2020).

### Other Antiviral Agents

It seems likely that a variety of other antiviral agents will be shown to have some effects on SARS-CoV-2 replication. Baloxavir inhibits the cap-dependent endonuclease, an essential enzyme for the initiation of mRNA synthesis of influenza viruses, thus preventing transcription of mRNA (Fukao et al., 2019). Tilorone, a synthetic small-molecule compound with antiviral activity, is proposed to induce interferon against pathogenic infection (Zhang, et al., 2015), which has been confirmed in Chikungunya virus (CHIK) and MERS-CoV was described *in vitro* (Ekins and Madrid, 2020). Recently, the activity against SARS-CoV-2 activity was shown in a Korean study (Jeon et al., 2020). Sofobuvir, an inhibitor of RdRp, was approved for treating Zika virus and hepatitis virus C(HCV) (Sacramento et al., 2017). It was also predicted to be effective against SARS-COV-2 RdRp as well based on the molecular insight that the HCV and the coronavirus share a similar viral genome replication mechanism (Ju et al., 2020). Alovudine, tenofovir and alafenamide, as RdRp inhibitors, could also have same potential against COVID-19 (Chien et al., 2020). More agents together with agents referred above which are promising in COVID-19 treatment are summarized in Table 1.

**Table 1:** Summary of potential agents used in the treatment of COVID-19.

Agents	Property	Mechanisms	References
NAbs	Antibody	Combine with surface epitopes of viral particles	Jeronimo et al. (2020)
rhACE2	Enzyme	Bind to ACE2 receptor	Zhang H et al. (2020)
Interferon antagonists	Protein	Inhibit excessive interferon	Channappanavar et al. (2016)
Baricitinib	JAK inhibitors	Restrain the JAK/STAT signaling pathway	Virtanen et al. (2019)
Ivermectin	Anti-parasitic	Inhibit importin-α/β-dependent nuclear transport	Lv et al. (2018)
Galidesivir	Adenosine analogue	Inhibiting viral RdRp	Warren et al. (2014)
Nelfinavir	Protease inhibitor	Inhibit viral main protease, Inhibit S protein-mediated membrane fusion	Yamamoto et al. (2004)
Baloxavir	Cap-dependent endonuclease inhibitor	Inhibit the cap-dependent endonuclease	Fukao et al. (2019)
Tilorone	Synthetic small-molecule compound	Induce interferon	Zhang et al. (2015)
Sofobuvir	Adenosine analogue	Inhibit viral RdRp function	Ju et al. (2020)
Natural killer cells	Innate immunity cell	Respond to viral infection without T cell help	Chen et al. (2010)
Fingolimod	S1P modulator	Prevent egress of lymphocytes from lymph nodes	Huwiler and Zangemeister-Wittke (2018)
Siponimod	S1P modulator	Prevent egress of lymphocytes from lymph nodes	Goodman et al. (2019)
Metronidazole	Antibiotic and antiprotozoal	Suppress cytokines storm	Gharebaghi et al. (2020)
Amantadine	Antiviral agent	Disrupt CTSL-mediated lysosomal pathway	Smieszek et al. (2020)
Teicoplanin	Antibiotic	Block endocytosis of virus	Baron et al. (2020)
Niclosamide	Anti-parasitic and anti-tumor	Block endocytosis and autophagy of virus	Pindiprolu and Pindiprolu (2020)
Minocycline	Antibiotics	Suppress cytokines storm	Alano et al. (2006) and Ge et al. (2020)
Triiodothyronine	Hormone	Promote the ability of natural killer cells	Pantos et al. (2020)
Melatonin	Hormone	Suppress cytokines storm	Li Y et al. (2020)

ACE2: angiotensin-converting enzyme two; CTSL: Cathepsin L; JAK/STAT: Janus kinase/signal transducer and activator of transcription; NAs: Neutralizing antibodies; RdRp: RNA-dependent RNA polymerase; rhACE2: recombinant human angiotensin-converting enzyme two; S1P: sphingosine one phosphate.

## Host system modulatory agents

Patients infected with SARS-CoV-2 have different clinical manifestations, with a range from asymptomatic to respiratory failure, multi-organ dysfunction. Better understanding of the pathogenesis facilitates proper management of COVID-19. Patients with severe respiratory failure are more likely to present sustainable TNF-α and IL-6 produced by circulating monocyte, which is distinct from bacterial sepsis or influenza (Giamarellos-Bourboulis et al., 2020). COVID-19 patients are also characterized by lower platelet count and lymphocytes, increased prothrombin time, D-dimer, and fibrin degradation products with aggravating disease (Di Minno et al., 2020; Liao et al., 2020; Tang N et al., 2020b). These coagulation abnormalities are reported to cause consequences ranging from venous embolism to DIC, or even death (Connors and Levy, 2020; Piazza et al., 2020). Immune dysregulation is reported to cause hyporeactive neutrophil and neutrophil extracellular traps, which interact with platelet and fibrin, contributing to microvascular thrombi in lung, kidney, and heart (Nicolai et al., 2020). Severe COVID-19 has a feature of an inflammatory signature, including high levels of inflammatory cytokines, alveolar inflammatory infiltrates, and vascular microthrombi, which leads to multi-organ failure (Chakraborty et al., 2020). Frustratingly, a WHO summary including most clinical trials and meta-analyses, concluded consistently these Remdesivir, HCQ, Lopinavir and interferon regimens mentioned above, appeared to have little or no effect on hospitalized COVID-19 (Pan et al., 2020). Due to absence of specific anti-viral agents and complicated pathogenesis of COVID-19, current treatment strategies focus on managing patients’ conditions.

### Corticosteroid

Corticosteroid, which makes contributions to inhibit cytokine storm (Figure 1F), is widely used in clinical practice for years and administrated during SARS and MERS epidemic, even though there are many divergences on the treatment effect and safety issues. In terms of COVID-19, the administration of corticosteroids has again been a conundrum for clinicians. On the one hand, early, low dose and short-term application of corticosteroids was associated with a faster improvement of clinical symptoms and absorption of lung foci in patients with severe COVID-19 pneumonia (Guzik et al., 2020). Also, low dose corticosteroid therapy did not delay viral clearance in patients with COVID-19 (Cao Y et al., 2020). An early short course of methylprednisolone in patients with moderate to severe COVID-19 reduced escalation of care and improved clinical outcomes (Fadel et al., 2020). A 7-days fixed-dose course of hydrocortisone or a shock-dependent dosing of hydrocortisone, favors days reduction for organ support (Angus et al., 2020). Corticosteroid treatment was associated with a lower risk of 30-days mortality, which was limited in the critically ill patients (Bartoletti et al., 2020). Despite the uncertain effect of corticosteroid therapy on overall survival, prudent dosing within effective limits may be recommended for critically ill patients under certain circumstances (Lu et al., 2020). Compared to standard use, high dose of corticosteroids (1–1.5 mg/kg/day) increased mortality exclusively in elderly patients and caused higher risk of mechanic ventilation requirement or death (Monreal et al., 2020). On the other hand, Wu et al. found that patients who received methylprednisolone treatment were much more likely to develop ARDS, probably because sicker patients were more likely to receive treatment, although methylprednisolone did appear to reduce the risk of death in patients with ARDS (Wu C et al., 2020). Corticosteroids impair the immune system, and current evidence does not support their use in lung injury (Russell et al., 2020). A meta-analysis showed that patients with severe conditions are more likely to require corticosteroids, but the use may lead to increased mortality and serious adverse reactions (Yang Z et al., 2020). Another study also showed no association between corticosteroid therapy and virus clearance time (Ding C et al., 2020), length of hospital stay or duration of symptoms (Jin et al., 2020). Short course use of methylprednisolone did not reduce mortality in the overall population with regard to a double-blind RCT (Jeronimo et al., 2020). Corticosteroid use showed no benefit in reducing in-hospital mortality for severe or critical cases, so the routine use of systemic corticosteroid among severe and critical COVID-19 patients was not recommended (Wu J et al., 2020). A RCT gives the conclusion that low-dose hydrocortisone didn’t significantly decrease death and duration of persistent respiratory support (Dequin et al., 2020).

In view of the current evidence and clinical experience, among adults receiving mechanical ventilation who do not have ARDS, routine use of systematic corticosteroids is advised against (weak recommendation, LQE). In those with ARDS, use of corticosteroids is advised (weak recommendation, LQE) (Poston, et al., 2020). For adults with COVID-19 and refractory shock, low dose corticosteroid therapy (“shock-reversal”), is recommended over no corticosteroid treatment. A typical corticosteroid regimen in septic shock is intravenous hydrocortisone (200 mg per day), administered either as an infusion or as intermittent doses (Alhazzani, et al., 2020). Large, well-designed clinical trials are needed to clarify the benefits of specific administration of corticosteroids in COVID-19.

### Convalescent Plasma and Immunoglobins

Neutralizing antibodies contained in CP and IVIG could bind to S1-RBD, resulting in limiting viral entry (Figure 1A). In COVID-19, CP is an undeniable choice for administration to patients for its specificity. Observational studies of patients in Wuhan showed that CP was an effective and specific therapy for COVID-19, which decreased viral load (Ye M et al., 2020). 80% recipients showed significant increase in antibody levels posttransfusion of CP in spite of variable titers from donors (Madariaga et al., 2020). When combined with systemic corticosteroids in severely ill patients, CP contributed to a reduction in viral load and caused no severe adverse effects (Ahn et al., 2020). As a conjunction to conventional therapy, CP speeded up being free of invasive mechanical ventilation support and elevated recovery rate (Gemici et al., 2020). Uncontrolled case series of patients, including a pregnant woman, recovered from COVID-19 after transfusion with CP (Chen X et al., 2020; Shen et al., 2020). Another study including 10 patients also got good outcomes and came up that one dose of 200 ml of CP derived from recently recovered donors with the neutralizing antibody titers above 1:640 is effective (Duan et al., 2020a). Duan et al. analyzed the feasibility of using CP in 19 patients and showed that one dose (200 ml) was well-tolerated and improved clinical outcomes (Duan et al., 2020b). A study in Texas indicated that administration of CP is a safe treatment option for those with severe COVID-19 disease, although the efficacy remained unclear (Salazar et al., 2020). Analysis of case series demonstrated CP was safe and might be efficacious as well (Pal et al., 2020). The transfusion of CP is safe in 5,000 hospitalized patients with COVID-19 based on early indicators, such as transfusion-associated circulatory overload (Joyner et al., 2020). Moreover, a multicenter retrospective cohort study in China, which recruited 325 critically ill adult patients from eight treatment centers, concluded that early administration of high dose IVIG significantly reduced mortality, decreased the inflammatory response and improved the function of some organs (Shao et al., 2020). In addition, a critically ill patient was cured successfully with plasma exchange followed by IVIG (Shi et al., 2020). Nevertheless, CP did not result in a statistically significant improvement in time to clinical improvement within 28 days (Li L et al., 2020). There was neither difference in risk of mortality or rate of hospital discharge between CP and control group (Rogers R et al., 2020), nor could progression to severe COVID-19 or all cause morality be ameliorated by CP (Agarwal et al., 2020). Recently, CP was reported to end SARS-CoV-2 shedding but not reduce the mortality rate in critically ill patients with end-stage COVID-19, which suggested treatment should be initiated earlier (Zeng et al., 2020). The optimal dose of CP or IVIG and time of administration needs further investigation in larger well-controlled trials to fully evaluate the clinical benefits.

### Neutralizing Antibodies (NAbs)

NAbs, which prevent viral attachment and accumulation, reduce infectivity by combining with surface epitopes of viral particles and blocking access of the virus to cells (Klasse, 2014). The constant region of the Ab can contribute to viral clearance through opsonization or complement activation, providing a highly specific immune defense (Coughlin and Prabhakar, 2012). Because SARS-CoV and SARS-CoV-2 both use ACE2 as an entry receptor (Tian X et al., 2020) and the receptor-binding domains (RBDs) of the two viruses are similar (Wan Y et al., 2020), NAbs against SARS may be effective in COVID-19 patients. Tian et al. (Tian X et al., 2020) recently showed that CR3022, a SARS-CoV NAb, binds to the RBD of SARS-CoV-2, although with an uncertain capability of neutralization. Some of the SARS-CoV-specific neutralizing antibodies that target the ACE2 binding site of SARS-CoV failed to bind 2019-nCoV spike protein, implying that the difference in the RBD of SARS-CoV and 2019-nCoV has a critical impact for the cross-reactivity of neutralizing antibodies. Bamlanivimab, a neutralizing IgG1 monoclonal antibody (mAb) directed against the spike protein of SARS-CoV-2, has been approved by FDA for treatment of mild-to-moderate COVID-19 in adult and pediatric patients (Coronavirus, 2020). A recent research even provided 11 potent human neutralizing antibodies for COVID-19 as therapeutic candidates (Wan J et al., 2020). Among NAbs isolated of from a convalescent patient, B38 and H4 block the binding between virus S-protein RBD and cellular receptor ACE2, displaying neutralization abilities. As for feasibility issues, NAbs is not only obtained from convalescent patients but also can be engineered in the laboratory (Zhao et al., 2015a; Zhao et al., 2015b; Li et al., 2017).

### Traditional Chinese Medicine

TCM has a long history and plays an indispensable role in the treatment of diseases because of its important roles in regulating immune system and inhibiting cytokine storm (Figure 1F). During the SARS epidemic in 2003, TCM was widely used in 58.3% of confirmed cases and achieved remarkable therapeutic effects. Based on previous experience of treating SARS with TCM, clinicians, especially in China, have encouraged the integrated use of TCM and Western medicine to treat COVID-19, and TCM has been included in the guidelines for COVID-19 treatment in China. Among 701 confirmed cases treated with *Qingfei Paidu* decoction (QPD), 130 cases were cured and discharged, clinical symptoms disappeared in 51 cases and improved in 268 cases, and symptoms remained stable, with no deterioration, in 212 cases (Publicity Department of the People’s Republic of China, 2020). Another investigation reviewed the results from four provincial hospitals in China that used QPD to treat 214 COVID-19 patients, taking three days as a course of treatment, and found that the total effective rate was >90%. The symptoms and imaging results of 60% of patients improved significantly and 30% of patients had stable symptoms without exacerbation (National Administration of Traditional Chinese Medicine, 2020). Another popular candidate, *Lianhuaqingwen* (LH), was shown to significantly inhibit SARS-CoV-2 replication in Vero E6 cells and markedly reduce production of pro-inflammatory cytokines (TNF-α, IL-6, CCL-2/MCP-1 and CXCL-10/IP-10) (Runfeng et al., 2020), suggesting that it might be a potential option for COVID-19 treatment. Several observational studies suggested that LH accelerated the disappearance of clinical symptoms, shortened the time for conversion to virus-negative status, and accelerated the improvement in chest CT scans (Cheng Deizhong et al., 2020; Lyu Ruibing and Li, 2020; Yao et al., 2020; Yi, 2020). A recent multicenter, prospective, RCT indicated LH achieved a higher recovery rate and a shorter recovery without reported adverse effects. Combination use of Lianhuaqingwen and umifenovir may accelerate recovery and improve the prognosis of patients with moderate COVID-19 (Fang et al., 2020). Another Chinese herbal extract, *Xuebijing*, also reduced the time for conversion to virus-negative status (Zhang C et al., 2020). Three cases from the same family, who received Western medicine combined with the Chinese traditional patent medicine *Shuanghuanglian* oral liquid, were reported to make a rapid recovery (Ni et al., 2020). *Tanreqing* capsule, significantly reduced the negative conversion time of fecal nucleic acid and the duration of negative conversion of pharyngeal-fecal nucleic acid (Zhang X et al., 2020). A retrospective study of four cases indicated that combination of Chinese and Western medicine improved the pneumonia-associated symptoms of COVID-19 (Wang Z et al., 2020). Both data mining of on-line databases and a core outcome set also concluded that TCM is effective for management of COVID-19 (Qiu et al., 2020; Zhou Z et al., 2020). Although high-quality evidence for the safety of some Chinese herbs is lacking (Luo et al., 2012), when used correctly based on patients’ situation, it is generally believed that there are no serious adverse reactions.

### Mesenchymal Stem Cells

Attention has been paid to the role of MSCs in attenuating the cytokine storm and suppressing overreaction of the immune system (Figure 1F). Clinical trials of different types of MSCs in COVID-19 patients are ongoing. Seven patients with SARS-CoV-2 pneumonia showed improved clinical outcomes without observed adverse effects after intravenous injection with bone marrow-derived MSCs for 14 days (Leng et al., 2020). MSC transplantation improved oxygen saturation for ARDS and increased the immune indicators, including CD4 and lymphocytes (Tang L et al., 2020). Treatment with adipose-derived stromal stem cells (ASCs) also shows promise in combating SARS-CoV-2 (Gentile and Sterodimas, 2020). 13 severe COVID-19 patients who were intravenously injected with ASC, mostly were extubated and discharged from ICU, with no significant adverse events (Sanchez-Guijo et al., 2020). Among stem cells, umbilical cord stem cells seem to be most desirable for treating SARS-CoV-2, because of noninvasive extraction procedures, fast doubling times and greater plasticity (Misra et al., 2020). Adoptive transfer therapy using human umbilical cord mesenchymal stem cells in a critically ill, 65-year-old patient with COVID-19, was well tolerated and led to a significant clinical improvement (Bing Liang et al., 2020). A phase one clinical trial revealed human umbilical cord mesenchymal stem cells was safe and well-tolerated by intravenous injection (Meng et al., 2020). Incidence of disease deterioration or severe complication of MSC treatment was rarely seen (Atluri et al., 2020). A novel technology for capturing the therapeutic properties of stem cells using nanotechnology has provided a new sight into MSC therapy (Metcalfe, 2020). Because of the complexities and ethical issues surrounding the use of MSCs, further clinical trials, with the highest standards of rational and appropriate design are needed (Khoury et al., 2020).

### Tocilizumab

It is well known that tocilizumab as a monoclonal antibody improving inflammatory condition by fighting against IL-6 (Figure 1F). A study in COVID-19 patients showed that intravenous administration of tocilizumab (8 mg/kg every 8 h) was highly beneficial (Michot et al., 2020). An Italian study also found that a patient who received tocilizumab (8 mg/kg every 12 h) for 2 days showed progressive improvements in both clinical condition and chest CT scans (Cellina et al., 2020). A single-dose use of tocilizumab, improved survival (Rossi B et al., 2020) and reduced lethality rate at 30 days with no significant toxicity in severe COVID-19 patients, who were without mechanical ventilation (Perrone et al., 2020). Response of COVID-19 pneumonia with ARDS to tocilizumab was rapid, sustained, and associated with significant clinical improvement, reduced mortality, and no obvious adverse reactions (Sciascia et al., 2020; Toniati et al., 2020; Xu X et al., 2020). It also shows short-term survival benefit in patients with severe COVID-19 illness (Ramaswamy et al., 2020). Treatment of a sickle cell patient infected with SARS-CoV-2 with tocilizumab and hydroxychloroquine led to a significant improvement in clinical condition (De Luna et al., 2020). In a preprint study, 30 selected patients showed that tocilizumab significantly reversed the cytokine storm and improved the condition of severely ill patients. The dosage of tocilizumab for COVID-19 patients can be determined based on those used to treat rheumatoid arthritis (Instruction of Tocilizumab, 2020). Time from lung injury onset to tocilizumab administration may be critical to patient recovery (Sanchez-Montalva et al., 2020). Early use lead to a positive impact during Covid-19 pneumonia with severe respiratory syndrome in terms of increased survival and favorable clinical course (Capra et al., 2020). Early stage administration of tocilizumab subcutaneously reduced the risk of death and improves clinical parameters, for example, CRP and lymphocyte counts (Malekzadeh et al., 2020). In addition to relieve hyper-inflammatory reaction, it is also beneficial for patients with liver dysfunction (Serviddio et al., 2020). Although tocilizumab group seemed to have improved survival outcome, these positive results need to be interpreted with caution since different research types and confounding factors (Wadud et al., 2020). Transient transaminitis was found to be the most common adverse reaction in patients 21 days post tocilizumab (Sirimaturos et al., 2020). However, no significant clinical improvement in temperature or oxygen requirements in most patients were observed in a US study (Rimland et al., 2020). A recent RCT concluded tocilizumab failed to prevent intubation or death in moderately ill hospitalized patients (Stone et al., 2020). Despite IL-6 receptor inhibitors might cause hypertriglyceridemia and acute pancreatitis (Morrison et al., 2020), tocilizumab is among the candidates for anti-inflammatory treatment in COVID-19.

### Other Anti-cytokines Therapeutics

Common anti-TNF agents, such as infliximab, adalimumab, thalidomid and golimumab (Mitoma et al., 2018), could be functional in inflammatory diseases. Common anti-TNF agents, such as infliximab, adalimumab, thalidomid and golimumab (Mitoma et al., 2018), could be functional in inflammatory diseases. A patient with IBD and COVID-19, was given adalimumab therapy, generated a quicker hospital discharge (Tursi et al., 2020). A patient with COVID-19 treated by thalidomide (Wang, 2020), got clinical improvements. Certainly, safety and efficiency need more support from clinical trials. Another anti-inflammation candidate, anakinra may be beneficial for treating severe COVID-19 patients with secondary HLH (Dimopoulos et al., 2020). Anakinra was used on nine consecutive severe COVID-19 pneumonia patients, and early chest CT scan showed the stopped extension of lesions. In this small open-label study, anakinra use was safe (Aouba et al., 2020). Although there is a shortage of relevant research about IL-1 receptor antagonists in COVID-19 patients, anakinra could potentially be beneficial in these patients. Additionally, Eculizumab, an agent that blocks the C5a pathway, should mitigate damage in COVID-19 patients and a 4-weeks study treatment with eculizumab did indeed remarkably improve the conditions of severe pneumonia or ARDS in COVID-19 patients, a finding that was supported by subsequent CT scans (Diurno et al., 2020). This discovery highlights a novel effective anti-inflammatory treatment, focusing on the complement system, which is worthy of further exploration as a treatment for COVID-19.

### Interferon Antagonists

Interferon, a glycoprotein with broad spectrum antiviral activity produced by innate immune cells, plays an important role in coronavirus infection (Hadjadj et al., 2020; Huang L et al., 2020; Volk et al., 2020). Interferon is a double-edged sword in viral diseases. On one hand, SARS-CoV encodes several proteins, including nsp13, nsp14, nsp15 and ORF6 (Yuen et al., 2020), that modulate innate immune signaling through the potential antagonism of the induction of interferon and by avoidance of interferon stimulated gene (ISG) effector functions (Totura and Baric, 2012). Downregulation of interferon expression assists SARS-CoV-2 infection because interferon is essential to prevent entry of coronaviruses into host cells (Volk et al., 2020). In addition, Interferon alfa-2a combined with ribavirin therapy is associated with significantly improved survival in MERS (Omrani et al., 2014). On the other hand, delayed IFN-I signaling promotes the accumulation of pathogenic inflammatory monocyte-macrophages (IMMs), resulting in elevated lung cytokine/chemokine levels, vascular leakage, and impaired virus-specific T cell responses in SARS-CoV-infected mice (Channappanavar et al., 2016). Early short-term blocking IFN-I after coronavirus infection evoked a long-lasting enhancement of immunological memory, which conferred improved protection upon subsequent reinfections (Palacio et al., 2020). Because excessive interferon responses during SARS-CoV-2 infection may lead to tissue damage (Zhou F et al., 2020), late phase interferon antagonist treatment should be considered.

### JAK Inhibitors

The JAK/STAT signaling pathway has been widely validated as a target for inflammation-related diseases (Virtanen et al., 2019). Moreover, Hadjadj et al. found an increase in peripheral blood of IL-6 and IL-6-induced genes, TNF-α and TNF-α pathway-related genes, as well as IL-10 (Hadjadj et al., 2020). JAK-STAT activation is also known to suppress the functions of granulocyte-macrophage colony-stimulating factor (McInnes et al., 2019). All of these indicated that JAK inhibitors could potentially be used to reduce inflammation in COVID-19 patients. JAK inhibitor curded activation of ACE2 and interferon-stimulated transcriptomes in human airway epithelium (Lee et al., 2020). Because JAK2 inhibition is reversible, transient treatment would not affect TH17 responses that are essential for innate immune responses and immunity against extracellular pathogens (Praveen et al., 2020). Baricitinib, Ruxolitinib and upadacitinib, JAK1/JAK2/JAK3 inhibitors, emerges as a potential agent (Richardson et al., 2020). Baricitinib stopped progression toward a severe/extreme form of the viral disease by restraining immune dysregulation in COVID-19 (Bronte et al., 2020). Ruxolitinib attenuated SARS-CoV-2 infection (Foss et al., 2020) and it rescued a patient who are refractory to anti-IL-6 therapy. Now, results of ongoing clinical trials will give a direction of JAK inhibitors administration.

### Combination of Agents

Some research indicated good outcome when oseltamivir used with other antiviral agents, like abidol, but the effect of oseltamivir alone remains unclear (Costanzo et al., 2020; Ding Q et al., 2020; Wang D et al., 2020). Moreover, oseltamivir has not been shown to have efficacy based on all investigation summary (Sanders et al., 2020). Furthermore, a retrospective study provided the first *in vivo* evidence that zinc sulfate in combination with hydroxychloroquine may play a role in therapeutic management for COVID-19 (Carlucci et al., 2020). This combination will be tested as a prophylactic regimen in a randomized clinical trial.

Treatment with IFN-α2b with or without umifenovir significantly reduced the duration of detectable virus in the upper respiratory tract and in parallel reduced duration of elevated blood levels for the inflammatory markers IL-6 and CRP (Liu et al., 2020). In COVID-19, triple combination of interferon beta-1b, lopinavir-ritonavir and ribavirin alleviated symptoms and shortened the duration of viral shedding and hospital stay in patients with mild to moderate COVID-19 (Hung et al., 2020). Besides, type III IFNs (IFN-λ) was believed to play an important role in SARS-CoV-2 and other viral infections (Prokunina-Olsson et al., 2020). Further application of interferon and combination regimen are undergoing more clinical trials.

### Anti-thrombotic Therapy

Rebalancing coagulation system, especially anti-platelet and anti-coagulant is crucial for COVID-19 coagulopathy administration. Antiplatelet therapy might be effective in improving the ventilation/perfusion ratio in COVID-19 patients with severe respiratory failure (Viecca et al., 2020). Aspirin, a classical anti-platelet agent, is possible to decrease mechanical ventilation rate, ICU admission and in-hospital mortality, without more bleeding events compared to non-aspirin use, based on evidence from a retrospective study (Chow et al., 2020). It is recommended that person suffering from SARS-CoV-2 infection should be administered with aspirin at the earliest (Haque et al., 2020). For the severe COVID-19 patients meeting SIC criteria or with markedly elevated D-dimer, using low molecular weight heparin seems to be associated with better prognosis (Tang N et al., 2020a). Dipyridamole, prohibiting platelet from aggregating, was shown to reduce viral replication, suppress hypercoagulability and enhance immune recovery, when taken as an adjunctive therapy in COVID-19 (Liu et al., 2020). Furthermore, longer duration of anti-coagulation was associated with reduced mortality risk (Paranjpe et al., 2020). Argatroban, a direct thrombin inhibitor, decreased further thrombosis complications (Arachchillage et al., 2020). Moreover, therapeutic-strength anticoagulation performed better than prophylactic anticoagulation without contributing to bleeding events (Boonyasai et al., 2020; Ferguson et al., 2020). Therapeutic anticoagulation is associated with a survival advantage among patients with COVID-19 who require mechanical ventilation in the ICU, as well (Trinh et al., 2020). Pre-admission applying anti-thrombotic, however, showed little protective effect in severe patients (Russo et al., 2020). COVID-19 patients receiving anti-coagulation medicine chronically, were prone to a higher mortality, resulting from cardiovascular events (Rossi R et al., 2020). A high incidence of venous thrombosis and worse outcome is observed, despite the use of heparin at the therapeutic dose (Pavoni et al., 2020). Routine chemical prophylaxis is believed to be inadequate in preventing venous thromboembolism in severe COVID-19, and different pharmacologic prophylaxis regimens are not helpful for lowering incidence of deep venous thrombosis (Maatman et al., 2020). High regimen thromboprophylaxis, like subcutaneous therapeutic unfractioned heparin, decreased the occurrence of pulmonary embolism (Taccone et al., 2020). Whether therapeutic or prophylaxis anti-thrombotic to be used, monitoring D-dimer is helpful for measuring efficiency and preventing adverse events (Song et al., 2020). Specific anticoagulation regimens may vary in different disease severity and need further determination based on clinical trials.

### Micronutrients Supplementation

Providing patients with sufficient nutrients through all stages of COVID-19 is also vital. Micronutrients, such as vitamin D, which is a modulator of adaptive immunity, may also be important. Serum concentrations of 25-hydroxy vitamin D tend to decrease with age (Vásárhelyi et al., 2011), and this may be associated with the severity of COVID-19 in the elderly (Novel, 2020). Moreover, vitamin D deficiency served as a predictor of high severity/mortality and poor prognosis in patients with acute respiratory failure due to COVID-19 (Carpagnano et al., 2020; Radujkovic et al., 2020), and as an indicator of high infection risk for the healthy (Merzon et al., 2020). Vitamin D is supposed to lower viral replication rates and reduce concentrations of pro-inflammatory cytokines via cathelicidins and defensins (Grant et al., 2020). Due to the underlying benefits, safety and low cost, it is rational to use it as a supplementary therapeutic in COVID-19. Vitamin C is also believed to significantly lower incidence of pneumonia based on three controlled trials with human subjects (Hemilä, 1997), which suggests it may affect susceptibility to lower respiratory tract infections under certain conditions (Hemilä, 2003). A clinical trial is undergoing to access the impact of high dose of vitamin C in patients with COVID-19 (Carr and Rowe, 2020), which is closed because of rare severe cases in Wuhan. Another micronutrient, vitamin K, its reduced level emerges as a potential risk factor of severe COVID-19 (Dofferhoff et al., 2020), suggesting supplement of vitamin K might be a necessary therapy.

## Conclusion

The ongoing public health crisis caused by SARS-CoV-2 is receiving massive global attention. Although several vaccines are being developed to protect against SARS-CoV-2, major efforts are underway to repurpose existing drugs to treat COVID-19. Herein, we have summarized current data, from both *in vitro* experiments and clinical research to address the effectiveness and safety of all candidates, applied to COVID-19 administration. CQ/HCQ inhibits virus infection via different stages, yet more risk instead of benefit is shown based on clinical studies. Remdesivir, due to the promising efficiency and more supportive evidence, is acknowledged as one of the therapeutic of COVID-19. In general, highly efficient anti-viral agents are absent in current treatment strategy. As for host system modulatory agents, use of corticosteroid and TCM need to be measured according to patients’ conditions, and the optimal dosage is uncertain. CP and MSC would theoretically provide passive immunity for patients and are relatively safe, however, they failed to achieve consistent results and are limited by the resources as well. Since it is well known that the incidence of thrombotic events is high, the strategy of therapeutic and prophylactic anti-thrombotic agents remains uncertain and needs to be taken into consideration. Lack of specific treatment for COVID-19 brings more attention to vaccine development to keep the pandemic in control and reduce severe condition. In summary, more large-scale randomized clinical trials are urgently required to provide high quality data and guide clinician to make better decision on treatment.
